# Interpolating Nonadiabatic Molecular Dynamics Hamiltonian with Bidirectional Long Short-Term Memory Networks

**DOI:** 10.1021/acs.jpclett.3c01723

**Published:** 2023-08-02

**Authors:** Bipeng Wang, Ludwig Winkler, Yifan Wu, Klaus-Robert Müller, Huziel E. Sauceda, Oleg V. Prezhdo

**Affiliations:** † Department of Chemical Engineering, University of Southern California, Los Angeles, California 90089, United States; ‡ Machine Learning Group, Technische Universität Berlin, 10587 Berlin, Germany; § Department of Chemistry, University of Southern California, Los Angeles, California 90089, United States; ∥ BIFOLD - Berlin Institute for the Foundations of Learning and Data, 10587 Berlin, Germany; ⊥ Department of Artificial Intelligence, Korea University, Anam-dong, Seongbuk-gu, Seoul 136-713, Korea; # Max Planck Institute for Informatics, Stuhlsatzenhausweg, 66123 Saarbrücken, Germany; 7 Google Deepmind, 10587 Berlin, Germany; 8 BASLEARN, BASF-TU joint Lab, Technische Universität Berlin, 10587 Berlin, Germany; 9 Departamento de Materia Condensada, Instituto de Física, Universidad Nacional Autónoma de México, Apartado Postal 20-346, 01000 México, D.F., México

## Abstract

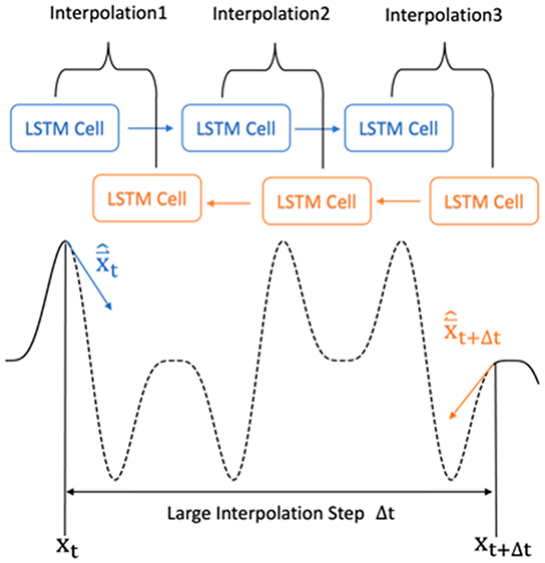

Essential for understanding far-from-equilibrium processes, nonadiabatic (NA) molecular dynamics (MD) requires expensive calculations of the excitation energies and NA couplings. Machine learning (ML) can simplify computation; however, the NA Hamiltonian requires complex ML models due to its intricate relationship to atomic geometry. Working directly in the time domain, we employ bidirectional long short-term memory networks (Bi-LSTM) to interpolate the Hamiltonian. Applying this multiscale approach to three metal-halide perovskite systems, we achieve two orders of magnitude computational savings compared to direct ab initio calculation. Reasonable charge trapping and recombination times are obtained with NA Hamiltonian sampling every half a picosecond. The Bi-LSTM-NAMD method outperforms earlier models and captures both slow and fast time scales. In combination with ML force fields, the methodology extends NAMD simulation times from picoseconds to nanoseconds, comparable to charge carrier lifetimes in many materials. Nanosecond sampling is particularly important in systems containing defects, boundaries, interfaces, etc. that can undergo slow rearrangements.

Nonadiabatic (NA) molecular dynamics (MD) serves as a potent computational tool for simulating excited state processes in a wide spectrum of chemical and physical systems.^1−8^ These simulations closely mirror time-resolved spectroscopy experiments, extensively used to characterize the ultrafast response of molecules and materials to external perturbations.^9−11^ The fusion of experimental and theoretical studies underpins numerous contemporary applications, including solar cells, light-emitting diodes, field-effect transistors, sensors, and quantum information devices. Implementing NAMD requires evaluation of geometry-dependent ground and excited states energies, and NA coupling (NAC) between the states.^12,13^ Such knowledge is typically obtained from computationally demanding ab initio electronic structure calculations performed alongside MD trajectories. To reduce the need for countless electronic structure calculations, methods such as classical path approximation (CPA) have been developed. Based on the observation that MD in many condensed matter and nanoscale systems is mostly influenced by thermal fluctuations rather than the occupied electronic state, the CPA substitutes multiple excited state trajectories with a single, typically ground state trajectory. However, even obtaining ground state MD trajectories at the ab initio level for large nanoscale systems over sufficient time scales remains computationally expensive. Hence, further advancements are necessary to enable atomistic modeling of complex modern materials and processes.

The pursuit of development of atomistic force fields (FF) to replace ab initio calculations and derive MD trajectories has come a long way, starting from simple physical models to incorporation of modern machine learning (ML) tools.^14,15^ Formulation of parametrized models for complex electronic properties,^16−18^ inclusive of excitation energies and NACs,^19−21^ has been impeded due to the high computational cost of ab initio calculations of such properties and scarcity of corresponding experimental data. Furthermore, NA Hamiltonians exhibit a markedly intricate relationship with system geometry, compared to the ground state energy and force, further complicating the task.

Ground state FFs, already generated routinely using ML techniques, enable the acquisition of lengthy, ab initio quality ground state trajectories. Instead of developing similar, geometry dependent ML models of excitation energies and NACs, we aim to sample these properties along a pregenerated MD trajectory, subsequently interpolating to derive the values for intermediate geometries. Though this approach may not be universally applicable in NAMD simulations, it promises significant computational savings under the CPA,^12,22,23^ especially in nanoscale systems where long-term fluctuations (100 ps or more) are crucial.^24−27^ The time-sampling/interpolation strategy can be adapted to NAMD simulations without the CPA, with ab initio calculations needed during the classical velocity rescaling, which is discussed, for instance, in ref (28). Our goal is to achieve an ML model of the NA Hamiltonian in nanoscale systems, bypassing the need for costly ab initio NAC calculations at every MD step for nanoseconds.

In this paper, we introduce a novel approach for predicting excitation energies and NACs for NAMD simulations using ML time-series interpolation. Employing Bidirectional Long Short-Term Memory networks (Bi-LSTM),^29^ we obtain a significant improvement over the previous methods, including inverse fast Fourier transform (iFFT),^30^ kernel ridge regression^31^ and neural networks.^32^ Well designed for time-interpolation, Bi-LSTM is a multiscale model that allows us to gain a two orders of magnitude savings compared to the direct ab initio calculation, as demonstrated with three metal-halide perovskite (MHP) systems that can undergo slow, large-scale atomic motions. Extremely popular for solar energy and optoelectronic applications, MHPs exhibit intricate MD with a wide range of anharmonic motions and time scales, posing challenges even for MLFF development. We obtain reasonable NAMD time scales for charge carrier trapping and recombination in MHPs by sampling the ab initio NA Hamiltonian only twice per picosecond and applying the Bi-LSTM network to fill in the intermediate values. In comparison, iFFT completely breaks down in such a regime. This innovative strategy allows us to substantially diminish the computational cost of ab initio quality NAMD calculations in modern nanoscale materials.

The quantum mechanical simulations, based on Density Functional Theory (DFT), were executed utilizing the Vienna Ab initio Simulation Package (VASP).^33−35^ The Perdew–Burke–Ernzerhof (PBE) density functional^36^ was deployed alongside projected augmented pseudopotentials.^37−39^ The cutoff energy for the plane wave basis was set at 450 eV, with the energy convergence criterion set to 10^–7^ eV. The ML-NADM approach was applied to three MHP systems, including pristine CsPbI_3_ and FAPbI_3_ (FA = formamidinium), and FAPbI_3_ containing a common replacement defect (FAI) that creates a midgap charge trap state. Models of pristine cubic CsPbI_3_, pristine tetragonal FAPbI_3_, and defective tetragonal FAPbI_3_ were constructed using simulation cells composed of 2 × 2 × 1, 2 × 2 × 2, and 2 × 2 × 2 unit cells, containing 20, 96, and 89 atoms, respectively. A FA moiety was replaced with an I atom in the pristine FAPbI_3_ system to create the FAI defect. The structures were visualized employing the VESTA software suite.^40^ Initially relaxed at 0 K, the structures were then heated to 300 K to obtain thermalized dynamics. Subsequently, 7 ps MD trajectories were generated in the microcanonical ensemble with 1 fs time step. The NAC calculation can be performed either analytically or numerically using infinitesimal increments in positions or time. In this study, the couplings were computed numerically by evaluating the overlap of wave functions at consecutive MD time steps, following the common practice under the CPA. The NA Hamiltonians obtained by time-series interpolation using the Bi-LSTM and iFFT methods are used to perform the NAMD simulations and to compare with the fully ab initio results. The sampled and interpolated NA Hamiltonians are iterated multiple times to obtain nanosecond NAMD results. The NAMD simulations are conducted using the decoherence-induced surface hopping (DISH) method,^41^ as implemented in the Pyxaid software.^12,13^ The pure-dephasing times are estimated using the second-order cumulant approximation of the optical response theory.^42^ 100 initial geometries are sampled from the microcanonical MD trajectories, and 1000 realizations of the stochastic DISH process are carried out for each initial geometry.

Figure 1 shows the optimized structures for pristine CsPbI_3_, pristine FAPbI_3_, and FAPbI_3_ with the FAI defect alongside their corresponding projected densities of states (DOS). Both pristine systems, CsPbI_3_ and FAPbI3, exhibit a direct bandgap between the valence band maximum (VBM) and the conduction band minimum (CBM), with the 0 K gaps of 1.67 and 1.35 eV, respectively. Introduction of the FAI defect generates an unoccupied electron trap state situated 0.45 eV below the CBM. In all three instances, the VBM is predominantly associated with I atoms, while the CBM is primarily supported by Pb atoms. The organic–inorganic systems exhibit a pronounced peak due to the organic FA groups, about 1.5 eV above the CBM. The trap state in the FAPbI_3_ perovskite with the FAI defect is localized around the I atom that substituted an FA, enabling efficient trapping of free electrons.

**Figure 1:**
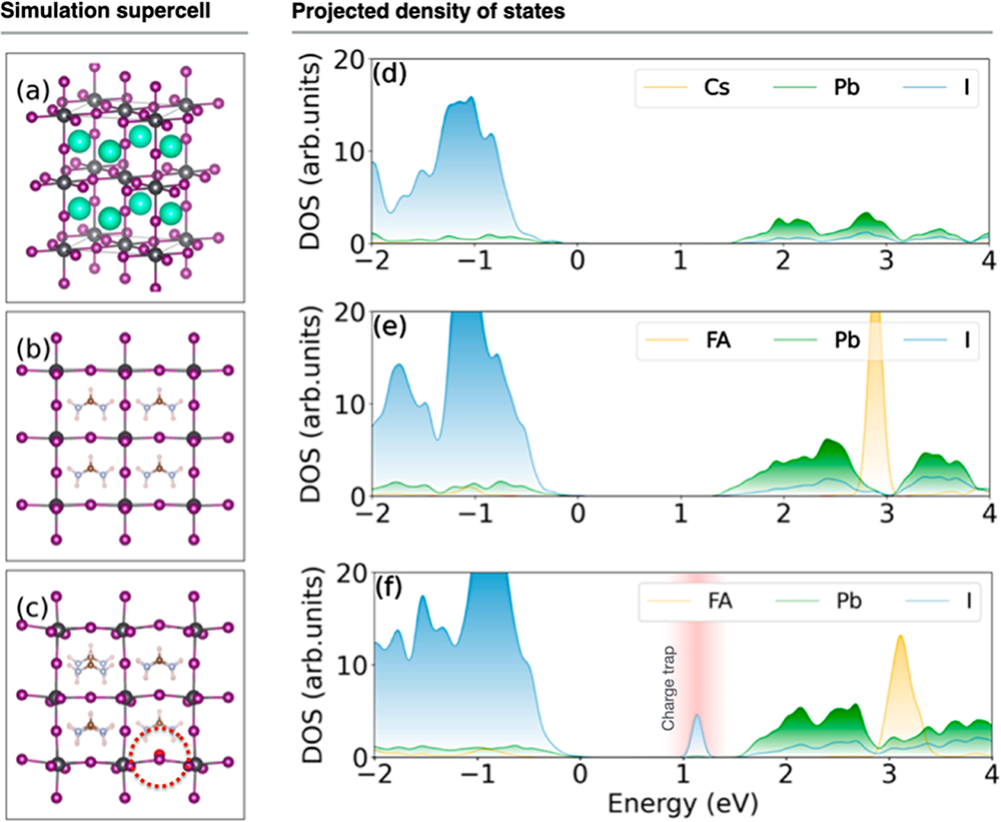
(a–c) Optimized structures of pristine CsPbI_3_, pristine FAPbI_3_, and FAPbI_3_ with the FAI defect and (d–f) projection of DOS, respectively. Energy reference is set to the VBM. In the defective system, a FA molecule is replaced by an iodine atom, which is marked red. A midgap electron trap is created by the defect.

Figure 2 depicts the iFFT-NAMD and Bi-LSTM-NAMD workflows. The details of the two methods can be found in ref (29) and ref (30), respectively. The iFFT interpolation of the NA Hamiltonian starts with using an FFT function to compute frequency data of the excitation energies and NAC. The data are sampled every 2^*n*^ timesteps, where *n* is an integer up to *n* = 9. The peak positions are retained, and the peak amplitudes are increased by a factor of 2^*n*^, to account for the data density. To obtain the excitation energies and NACs for the intermediate times by iFFT interpolation, we create frequency information matching the full data set length. We apply “zero padding” to the sampled amplitude, extending the short, sampled frequency range to match the full trajectory. Finally, we perform iFFT to the zero-padded amplitude and obtain the interpolated energy and NAC values.

**Figure 2:**
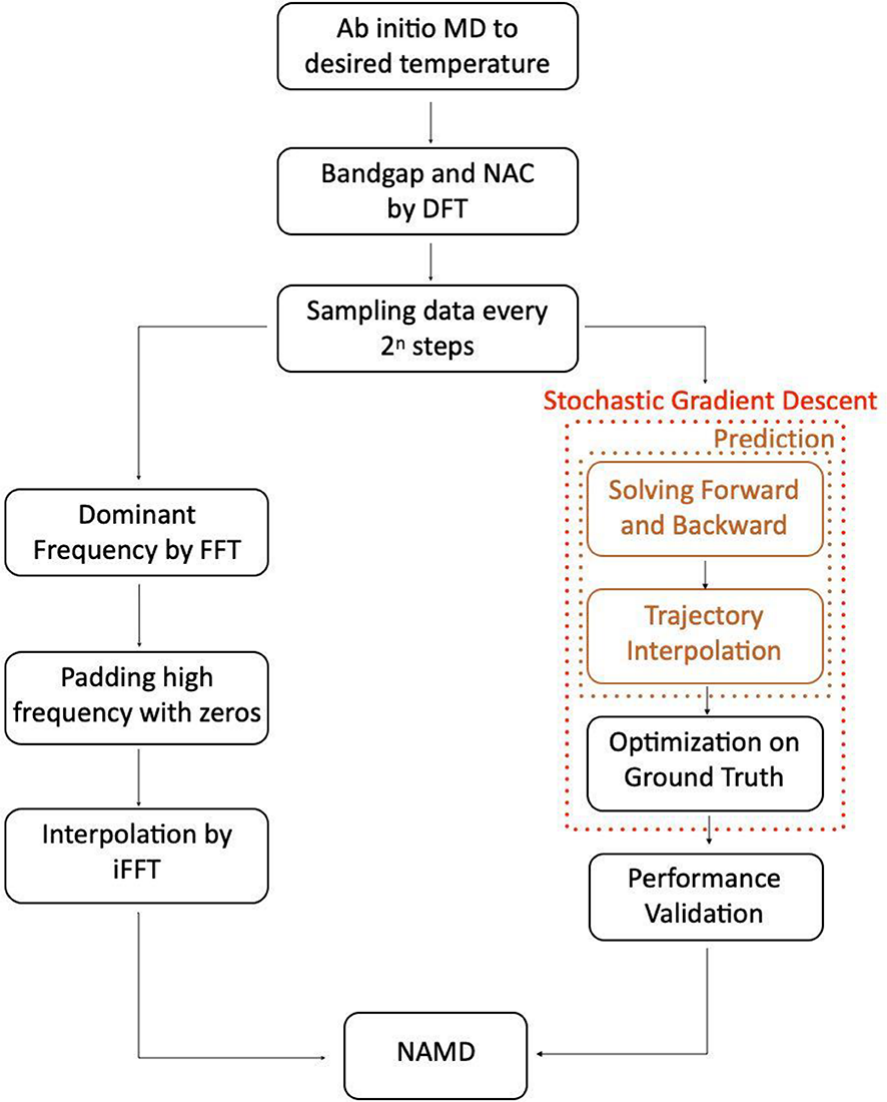
Schemes of the iFFT and Bi-LSTM methods. The left side gives the iFFT-NAMD workflow, while the right side presents the Bi-LSTM-NAMD flow.

The Bi-LSTM-NAMD workflow, Figure 2 (right), employs a slightly more complex strategy. Using the PyTorch library,^43^ we implement a Bi-LSTM model, which utilizes two LSTM networks that process data from the opposite directions, thus incorporating past and future inputs for a comprehensive data analysis. The excitation energies and corresponding NACs serve as inputs for the Bi-LSTM model, which comprises predefined Bi-LSTM units in hidden layers. The trained Bi-LSTM model can predict the unsampled sequence points, eliminating the need for frequency analysis or the zero padding required in the iFFT approach.

Figure 3 displays the final 20% (approximately 1.5 ps) of the ab initio energy gaps and NACs, along with predictions from both the Bi-LSTM and iFFT models for the VBM, CBM, and trap states in the pristine and defective systems. We utilized interpolation steps of 64 and 128 fs. In these instances, the first 80% of the ab initio data serve as the training data set, from which sequences of 64 and 128 steps are randomly extracted. The Bi-LSTM uses the first and last step of these sequences as boundary conditions to reconstruct the entire sequence of length 64, respectively 128. The model is evaluated in a similar fashion. When used for predictions, the model reconstructs complete sequences while being provided with 1.6% and 0.8% of evenly sampled steps. Since iFFT does not require model training, it directly samples 20% of the data at intervals of 64 and 128 steps and analyzes its frequency information to interpolate the energy gaps and NACs. Both Bi-LSTM and iFFT accurately replicate the peaks in the energy gaps and NACs. However, Bi-LSTM outperforms iFFT in maintaining fast oscillations, even when using larger interpolation steps. This is attributed to the model’s utilization of integration steps smaller than the interpolation step as encoded in its loss function, enabling a higher resolution by interpolating between two subsequent phase spaces. Furthermore, Bi-LSTM exploits its memory blocks to capture local and nonlocal correlations in the time series, hence reconstructing the high-frequency spectrum hidden in the data.^29^ In contrast, iFFT loses more oscillation information due to “zero padding”, which substitutes high frequencies in the frequency domain with zeros. This phenomenon is rooted in the fact that the slow movement of heavy lead and halide atoms in lead halide perovskites largely dictates the MHP vibrational motions that couple to the electronic subsystem. With interpolation steps of 64 and 128, both iFFT and Bi-LSTM perform well. However, previous research indicates that fast oscillations are less impactful on NAMD time scales, prompting us to test the models with larger interpolation steps. Determining the upper limit for interpolation steps would aid in balancing computational resource savings and the data recovery speed.

**Figure 3:**
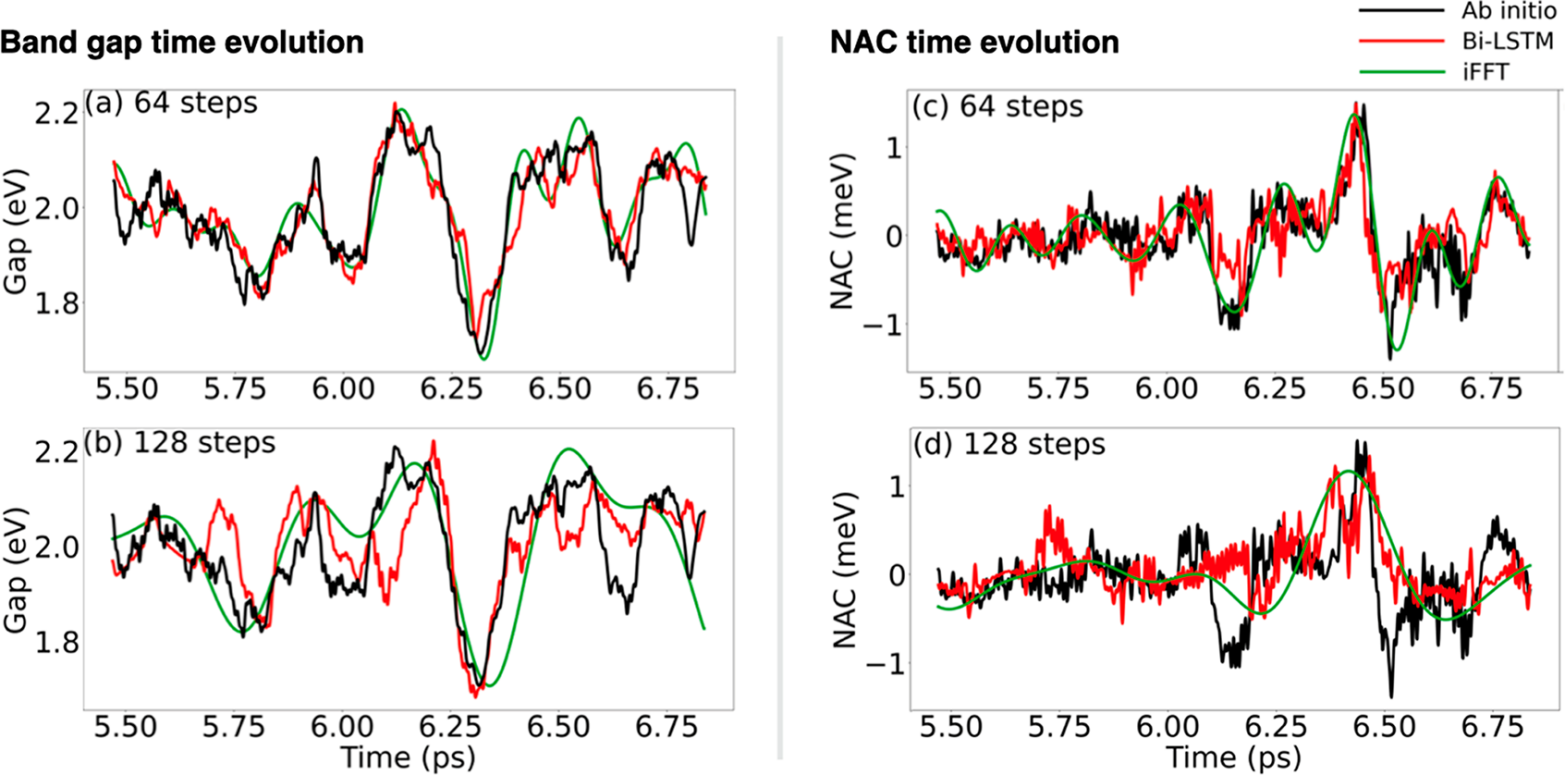
(a,b) Bandgap and (c,d) NAC of pristine FAPbI_3_ obtained by the iFFT and Bi-LSTM methods by sampling the ab initio data every 64 and 128 steps. The iFFT method captures only the slow envelope fluctuations, while the Bi-LSTM model represents the fast oscillations, which are particularly pronounced in the NAC.

Figure 4 illustrates the prediction capabilities of the Bi-LSTM and iFFT models when dealing with a substantial increase in the interpolation step set to 512 fs (0.2%). The Bi-LSTM model retains the ability to capture most oscillation peaks, whereas the iFFT model can only roughly approximate the fluctuation. The inadequate iFFT representation stems from the fact that the frequency associated with such a large interpolation step surpasses even the slow motions of the MHP inorganic lattice. “Zero padding” expands from the high-frequency area into the medium-frequency range, leading to the loss of significant oscillation information. The FTs of the energy gaps shown in Figure 5 confirm that Bi-LSTM is a multiscale model, capturing the whole range of frequencies, in contrast to iFFT that represents only very low frequencies. However, even under these challenging conditions, both Bi-LSTM and iFFT models still produce decent predictions of averaged energy gaps, as depicted in Table 1. Note that electronic energy levels in MHPs and other systems exhibit fewer fluctuations compared to NACs. Given the complex dependence of NAC on atomic geometries, discerning the NAC patterns with only 0.2% data presents a greater challenge.

**Table 1:** Canonically Averaged Energy Gap, Average Absolute NAC, and Charge Recombination Times in Pristine CsPbI_3_, Pristine FAPbI_3_, and Defective FAPbI_3_^a^

	**Avg Gap (eV)**			**Avg Abs NAC (meV)**		
	CsPbI_3_	FAPbI_3_	Defective FAPbI_3_	CsPbI_3_	FAPbI_3_	Defective FAPbI_3_
Ab initio	1.85	1.98	1.14	0.45	0.29	0.28
Bi-LSTM	1.91	2.05	1.29	0.58	0.36	0.20
iFFT	1.87	2.01	1.26	0.48	0.44	0.14

	**Recombination Times (ns)**		
	CsPbI_3_	FAPbI_3_	Defective FAPbI_3_
Ab initio	85	60	65
Bi-LSTM	64	66	21
iFFT	161	255	15439

a The data for defective FAPbI_3_ correspond to the trap-VBM transition, which constitutes the bottleneck and determines the recombination time. The ab initio sampling is performed every 512 fs.

**Figure 4:**
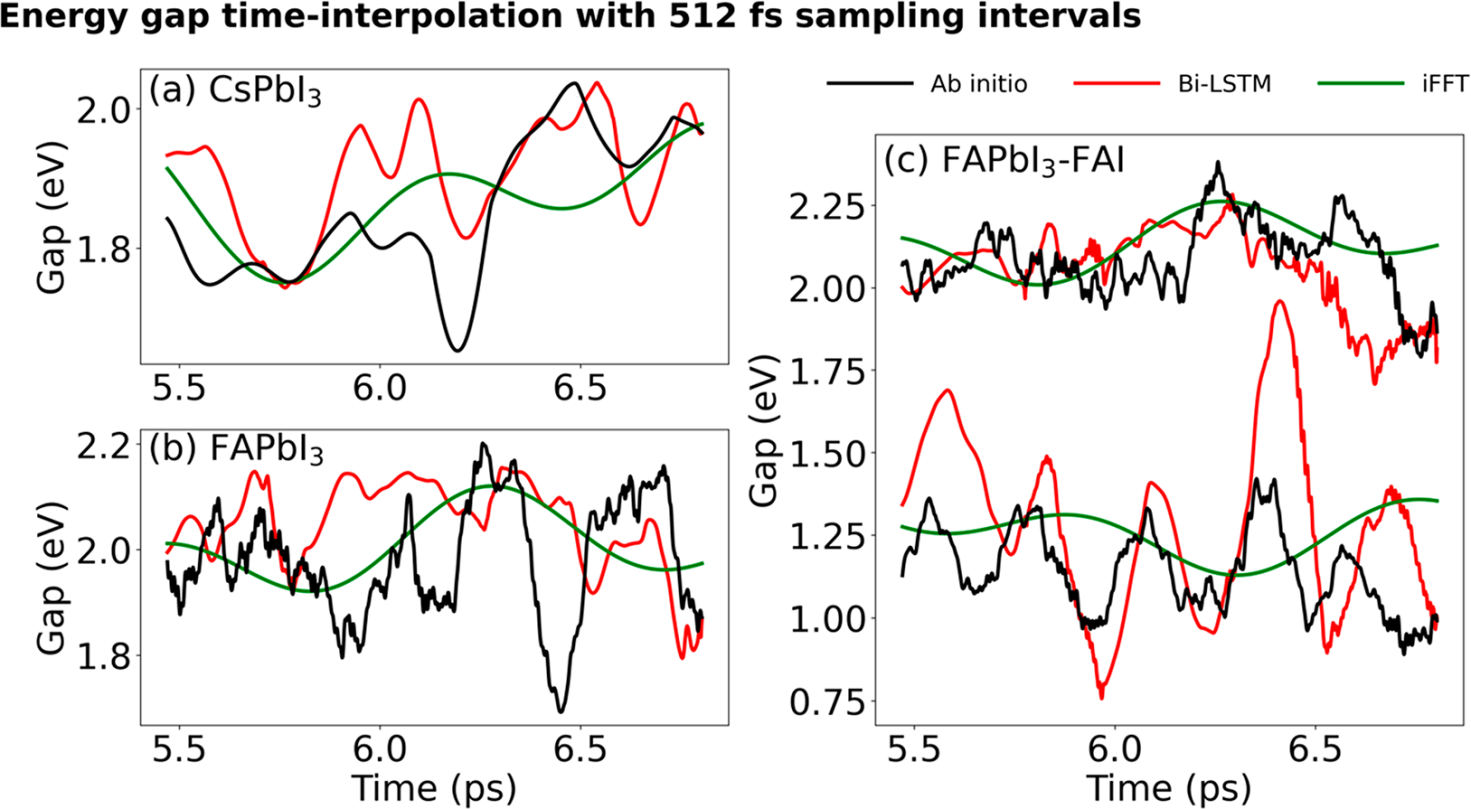
Energy gaps calculated by the ab initio and Bi-LSTM and iFFT models. The data are sampled every 512 fs, creating a very significant computational saving. (a,b) Gaps of pristine CsPbI_3_ and FAPbI_3_. (c) Gaps of defective FAPbI_3_. The gap of around 1 eV corresponds to the VBM-trap, while the gap around 2 eV corresponds to the VBM-CBM, Figure 1f. The Bi-LSTM model is able to capture most of the oscillation peaks, while the iFFT method represents the gap fluctuations only roughly.

**Figure 5:**
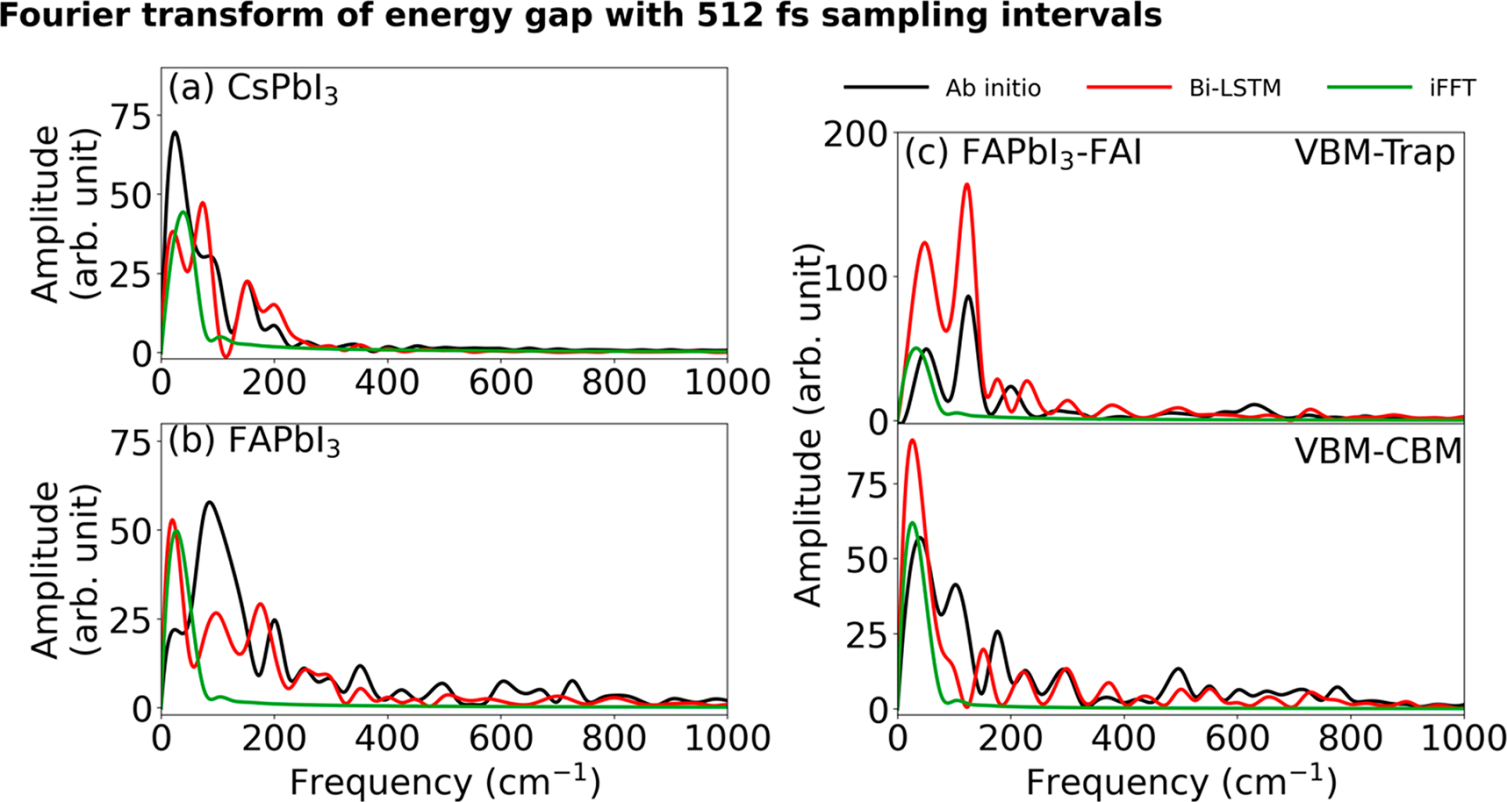
FTs of the energy gaps calculated by the ab initio, Bi-LSTM, and iFFT models with a 512 fs interpolation step for (a) pristine CsPbI_3_, (b) pristine FAPbI_3_, and (c) defective FAPbI_3_. The Bi-LSTM model captures both slow and fast scales, while iFFT gives low frequencies only.

Figure 6 presents the NAC predictions from the Bi-LSTM and iFFT models with an interpolation time of 512 fs. Both interpolation models only provide a coarse representation of the NAC. However, the Bi-LSTM model manages to capture the fast oscillations to some degree, while the iFFT model merely displays mildly curved waves. The inadequate recovery of the ab initio data by the iFFT model is due to the scarce frequency information in the sampled data points (only 0.2%, 3 points in a 1.5 ps trajectory). Thus, we encounter accuracy limitations of the iFFT model when processing complex defective organic perovskites. Conversely, the Bi-LSTM model demonstrates its capabilities in managing large interpolation steps. By calculating derivatives of the ab initio data from opposing directions, Bi-LSTM exhibits outstanding error control for very large interpolation steps. The Bi-LSTM prediction not only identifies the large, slow fluctuations arising from the heavy atoms but also reproduces the fast oscillations induced by the organic groups.

**Figure 6:**
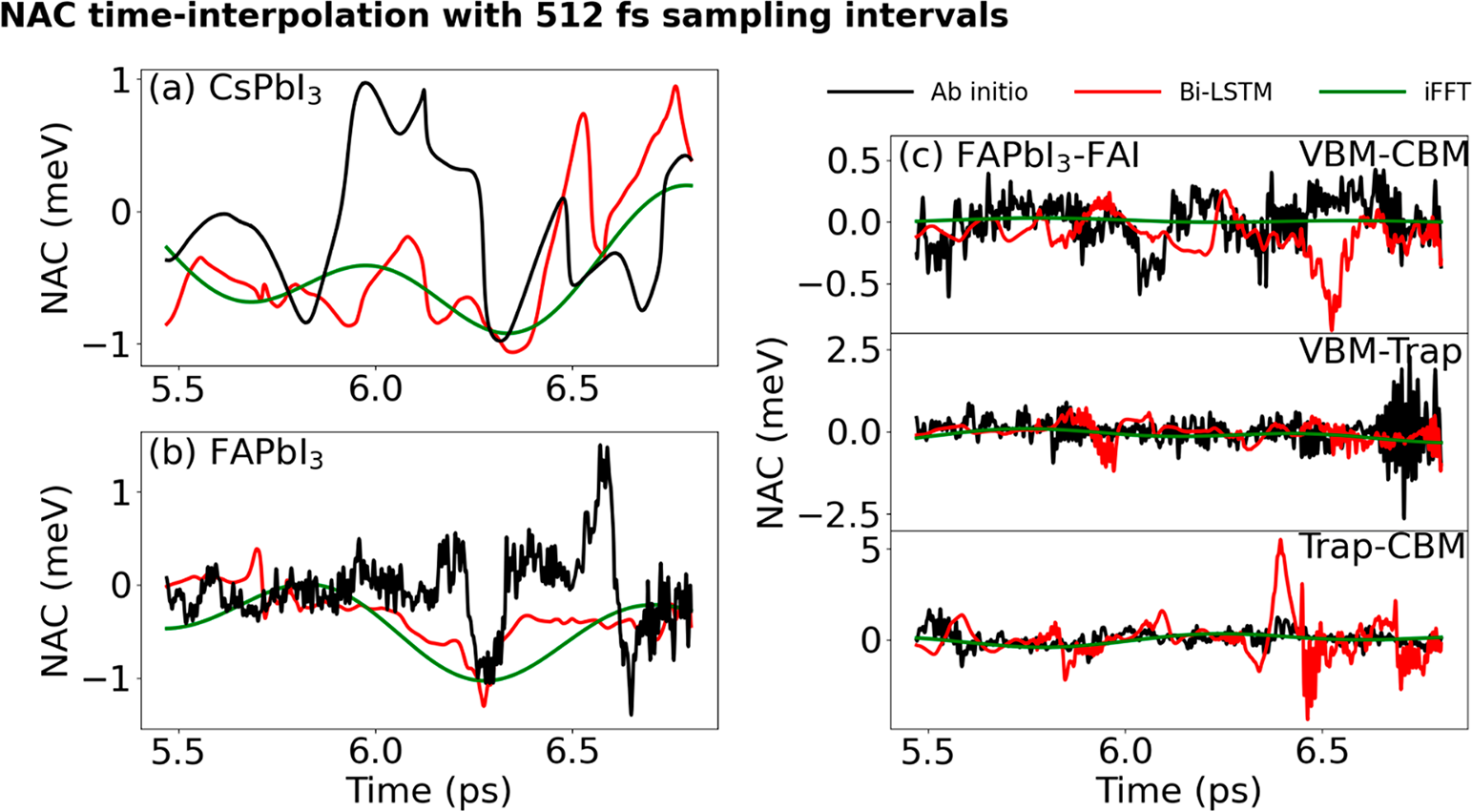
NACs calculated by the ab initio, Bi-LSTM, and iFFT models. The data are sampled every 512 fs, creating a very significant computational saving. (a–c) NACs of pristine CsPbI_3_, pristine FAPbI_3_, and defective FAPbI_3_, respectively. Both models provide a crude description of the NAC. The Bi-LSTM model is able to capture the fast oscillations. The iFFT method represents the overall NAC fluctuations in pristine CsPbI_3_ and FAPbI_3_. However, it has exceeded its accuracy limit for defective FAPbI_3_: the green lines in part (c) hardly deviate from 0.

Table 1 collects the essential statistics of the predictions. Remarkably, most values are predicted quite well by both Bi-LSTM and iFFT even though the ab initio data are sampled once every 512 fs. More detailed statistics are presented in Tables S1–S3. The VBM-Trap NAC is the most challenging property to predict, while it is critical since the VBM-Trap transition forms the bottleneck and governs the overall recombination time when the defect introduces an electron trap. Average absolute values of the Trap-VBM NAC for the ab initio, Bi-LSTM, and iFFT methods in FAPbI_3_ with the FAI replacement defect are 0.28, 0.20, and 0.14 eV, respectively. The substantial reduction of the NAC significantly slows down the electron–hole recombination and leads to inaccurate results in the NAMD calculations.

Figure 7 presents the NAMD simulation results derived from the ab initio, Bi-LSTM, and iFFT Hamiltonians, highlighting the rise or decay of populations in the key states during electron–hole recombination dynamics. For pristine CsPbI_3_ and FAPbI_3_, the ground state population grows from zero to a maximum of 0.15 within 10 ns, indicating up to a 15% probability of electron–hole recombination within 10 ns. In both cases, even with the large interpolation step, the Bi-LSTM model yields results closer to the ab initio method than the iFFT model. The recombination times for the ab initio method for pristine CsPbI_3_ and FAPbI_3_ are 85 and 60 ns, respectively. The recombination times for the Bi-LSTM and iFFT models are 64 and 66 ns and 161 and 255 ns, respectively, Table 1. The results from the Bi-LSTM show a relatively minor discrepancy from the ab initio method than the iFFT model. In the defective system, the Bi-LSTM-NAMD calculations suggest that charge trapping occurs more rapidly than in the ab initio NAMD. This effect is due to Bi-LSTM’s overestimation of the significant fluctuation at the CBM-Trap NAC, as depicted in Figure 5c. The iFFT results do not show noticeable recombination within the NAMD time scale, which can be attributed to an unreasonable underestimation of the NACs for all three states in the defective system. The recombination times for the ab initio, Bi-LSTM, and iFFT methods are 65, 21, and 15,439 ns, respectively. Notably, even with only 0.2% data sampling (3 geometries per 1.5 ps), the Bi-LSTM still provides a reasonably accurate estimate for the charge carrier lifetime in the defective system, while the iFFT model produces an implausible estimation. As sampling increases, both the Bi-LSTM and iFFT results converge with the ab initio results, as shown in Figure 3. Overall, the Bi-LSTM-NAMD method provides an acceptable estimation of the charge trapping and recombination times in both all-organic and mixed organic–inorganic MHPs perovskites, with a defect, using a mere ∼0.2% of the ab initio data, i.e., with the data sampling only once every 0.5 ps.

**Figure 7:**
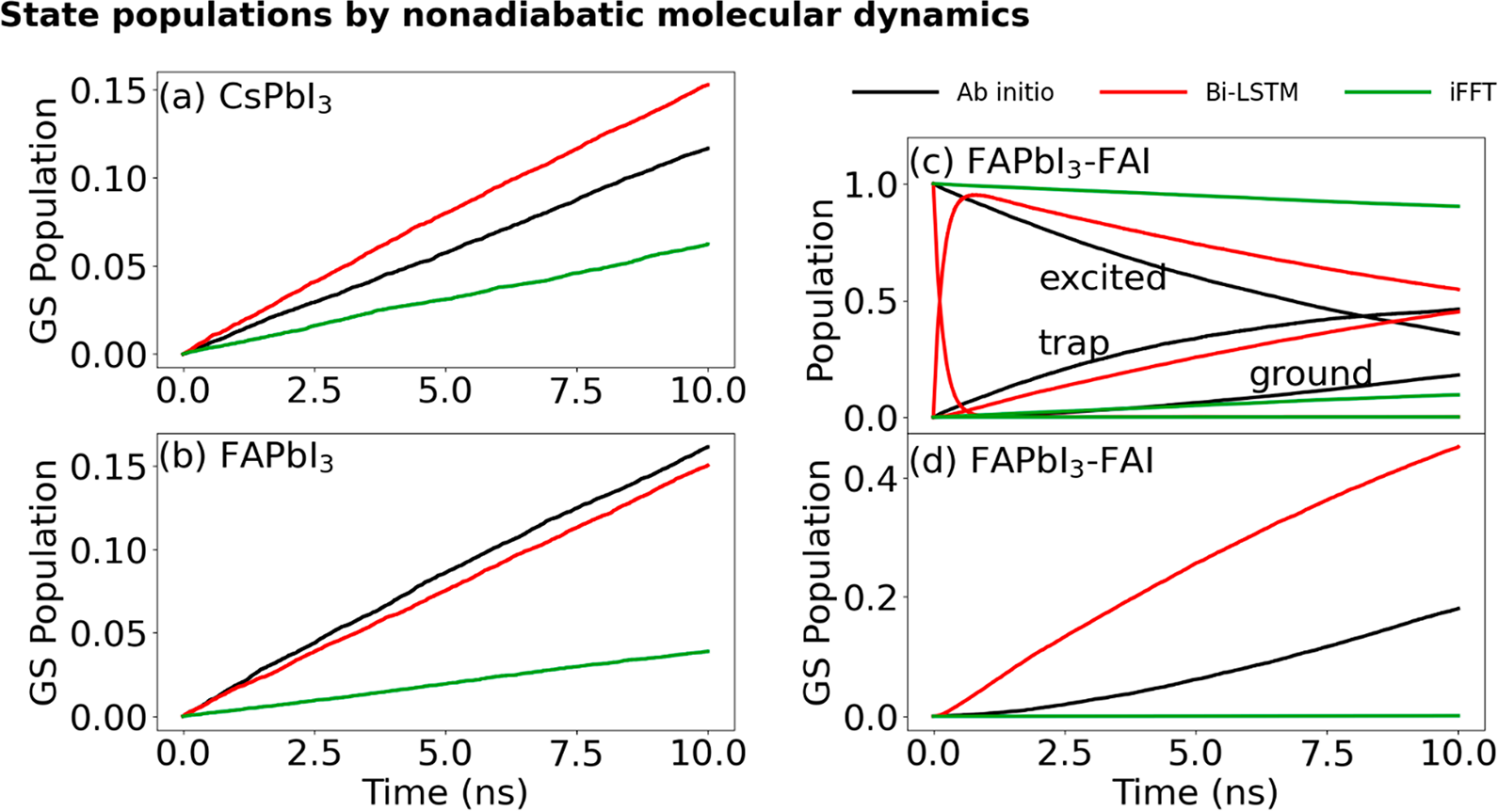
(a,b,d) Ground state population of pristine CsPbI_3_, pristine FAPbI_3_ and defective FAPbI_3_ obtained by NAMD calculations. (c) Populations of excited, trap, and ground states in defective FAPbI_3_. The corresponding time scales are presented in Tables 1 and S1–S3. The Bi-LSTM model provides a significant improvement over the iFFT scheme. Importantly, even with the data sampling of every 512 fs, the Bi-LSTM model reproduces the ab initio charge recombination times within a factor of 3. The iFFT method gives good results for the pristine systems; however, it breaks down for defective FAPbI_3_, because the complex fluctuation of the NAC causes a breakdown of the iFFT method with such a sparce sampling.

In summary, we have developed a Bi-LSTM time-series interpolation scheme of the NA Hamiltonian for use in ab initio quality NAMD simulations and compared the developed methodology with the fully ab initio and iFFT interpolation methods. We tested the methodology with all-inorganic and hybrid organic–inorganic MHPs containing a common defect. MHPs exhibit complex long-time geometric evolution that influences their electro-optical and charge carrier properties, providing an important application to a modern and extensively studied material. Both iFFT and Bi-LSTM models reproduce well phonon-induced fluctuations in the energy gaps and NACs with 64 and 128 fs interpolation steps, but being a multiscale model, Bi-LSTM demonstrated an advantage in capturing fast oscillations arising from the inorganic FA subsystem. This is mainly due to the Bi-LSTM capacity to handle data from both past and future, which enables capturing local and nonlocal correlations in the time series, providing a higher resolution of intermediate data points. While the iFFT model works well for lower frequency oscillations dominated by slow-moving heavy atoms, it fails to capture higher frequency oscillations from the organic groups due to inherent limitations of the zero-padding frequency domain analysis. When the sampling step is increased to 512 fs, the Bi-LSTM model outperforms the iFFT model even more notably. In particular, the iFFT method completely fails with the defective system, while the Bi-LSTM model still gives reasonable charge trapping and recombination times. This is because localized trap states exhibit faster local vibrational motions that cannot be captured by iFFT with such a large interpolation step. It is quite remarkable that the developed Bi-LSTM-NAMD method can produce reasonable estimates of charge carrier trapping and recombination times in MHPs with the ab initio data sample as rarely as twice per picosecond. Such sampling provides more than two orders of magnitude in computational savings compared to the direct ab initio NAMD simulation. The study paves the way for a deeper understanding of electron dynamics in complex nanoscale materials that can undergo slow structural motions requiring proper long-time sampling. In combination with an ML-FF, the Bi-LSTM-NAMD methodology significantly reduces the computational cost of ab initio quality quantum dynamics simulations, extending the simulation time from pico- to nanoseconds.

## Supporting Information Available

Ab initio and machine learning nonadiabatic Hamiltonian and inelastic and elastic electron-vibrational scattering times (PDF)
