# Supplementary material for: Interpolating Nonadiabatic Molecular Dynamics Hamiltonian with Bidirectional Long Short-Term Memory Networks
Source: J Phys Chem Lett. 2023 Aug 2;14(31):7092–9. doi: 10.1021/acs.jpclett.3c01723 (PMC10424239; doi:10.1021/acs.jpclett.3c01723)
Supplement: Supplementary file 1 — jz3c01723_si_001.pdf [file jz3c01723_si_001.pdf]

Supporting Information for:

Interpolating Nonadiabatic Molecular Dynamics Hamiltonian with Bi-directional Long Short-Term Memory Networks

Bipeng Wang,[1] Ludwig Winkler,[2] Yifan Wu,[3] Klaus-Robert Müller,[2,4,5,6,7] Huziel E. Sauceda,[2,8,9] Oleg V. Prezhdo[1,3,*]

[1]*Department of Chemical Engineering, University of Southern California, Los Angeles, CA 90089, USA*

[2]*Machine Learning Group, Technische Universität Berlin, 10587 Berlin, Germany*

[3]*Department of Chemistry, University of Southern California, Los Angeles, CA 90089, USA*

[4]*BIFOLD - Berlin Institute for the Foundations of Learning and Data, Berlin, Germany*

[5]*Department of Artificial Intelligence, Korea University, Anam-dong, Seongbuk-gu, Seoul 136-713, Korea*

[6]*Max Planck Institute for Informatics, Stuhlsatzenhausweg, 66123 Saarbrücken, Germany*

[7]*Google Research, Brain team, Berlin, Germany*

[8]*BASLEARN, BASF-TU joint Lab, Technische Universität Berlin, 10587 Berlin, Germany*

[9]*Instituto de Física, Universidad Nacional Autónoma de México, Apartado Postal 20-346, 01000 México, D.F., México*

[*] Corresponding author. Email: prezhdo@usc.edu

Table S1. Canonically Averaged (Mean) Energy Gap, Root Mean Square (RMS) Gap, Average Absolute NAC, Root Mean Square NAC, Recombination Time and Pure-Dephasing Time of the Ab Initio, Bi-LSTM and iFFT Models for Pristine $CsPbI_3$. The ab initio sampling is performed every 512 fs.

**Pristine $CsPbI_3$**	**Gap (eV)**		**Abs NAC (meV)**	
	Mean	RMS	Mean	RMS
Ab initio	1.851	1.854	0.450	0.527
Bi-LSTM	1.907	1.908	0.577	0.629
iFFT	1.859	1.860	0.538	0.588
	**Recombination Time (ns)**		**Pure-Dephasing Time (fs)**	
Ab initio	85		6.6	
Bi-LSTM	64		8.5	
iFFT	161		10.0	

Table S2. Canonically Averaged (Mean) Energy Gap, Root Mean Square (RMS) Gap, Average Absolute NAC, Root Mean Square NAC, Recombination Time and Pure-Dephasing Time of the Ab Initio, Bi-LSTM and iFFT Models for Pristine $FAPbI_3$. The ab initio sampling is performed every 512 fs.

**$FAPbI_3$ pristine**	**Gap (eV)**		**Abs NAC (meV)**	
	Mean	RMS	Mean	RMS
Ab initio	1.980	1.984	0.290	0.408
Bi-LSTM	2.051	2.053	0.357	0.432
iFFT	2.008	2.009	0.448	0.553
	**Recombination Time (ns)**		**Pure-Dephasing Time (fs)**	
Ab initio	60		6.1	
Bi-LSTM	66		8.1	
iFFT	255		11.2	

Table S3. Canonically Averaged (Mean) Energy Gap, Root Mean Square (RMS) Gap, Average Absolute NAC, Root Mean Square NAC, Recombination Time and Pure-Dephasing Time of the Ab Initio, Bi-LSTM and iFFT Models for Defective $FAPbI_3$. The ab initio sampling is performed every 512 fs.

**$FAPbI_3$ FAI**	**Gap (eV)**				**Abs NAC (meV)**					
	Trap		CBM		VBM-Trap		VBM-CBM		Trap-CBM	
	Mean	RMS	Mean	RMS	Mean	RMS	Mean	RMS	Mean	RMS
Ab initio	1.144	1.151	2.088	2.091	0.281	0.415	0.147	0.183	0.317	0.426
Bi-LSTM	1.291	1.318	2.056	2.059	0.197	0.276	0.140	0.190	0.733	1.111
iFFT	1.252	1.254	2.131	2.132	0.113	0.136	0.011	0.015	0.182	0.229

	**Recombination Time (ns)**	**Pure-Dephasing Time (fs)**		
		VBM-Trap	VBM-CBM	Trap-CBM
Ab initio	65	5.4	6.1	4.7
Bi-LSTM	21	2.5	5.5	2.3
iFFT	15439	9.8	9.2	5.1
